# Participatory System Dynamics Approach Targeting Childhood Health in a Small Danish Community (Children’s Cooperation Denmark): Protocol for a Feasibility Study Design

**DOI:** 10.2196/43949

**Published:** 2023-03-07

**Authors:** Knud Ryom, Helene Kirkegaard, Steven Allender, Anna Aaby, Christina Breddam, Helle Terkildsen Maindal, Jane Nautrup Østergaard

**Affiliations:** 1 Department of Public Health Aarhus University Aarhus Denmark; 2 Steno Diabetes Centre Aarhus Aarhus University Hospital Aarhus Denmark; 3 Institute for Health Transformation Deakin University Melbourne Australia; 4 Health, Culture and Care Randers Municipality Randers Denmark

**Keywords:** system dynamics, community-based participatory research, feasibility, childhood health, community, design, acceptability, intervention, evaluation, implementation, effectiveness, testing

## Abstract

**Background:**

Improving childhood health is complex due to the multifactorial nature and interaction of determinants. Complex problems call for complex intervention thinking, and simple one-size-fits-all solutions do not work to improve childhood health. Early awareness is important, as behavior in childhood often is manifested across adolescence and into adulthood. To facilitate shared understanding of the complex structures and relationships that determine children’s health behavior, participatory system approaches in, for example, local communities have shown promising potential. However, such approaches are not used systematically within public health in Denmark, and before being rolled out, they should be tested for their feasibility within this context.

**Objective:**

This paper describes the study design for Children’s Cooperation Denmark (Child-COOP) feasibility study that is aiming to examine the feasibility and acceptability of the participatory system approach and the study procedures for a future scale-up controlled trial.

**Methods:**

The feasibility study is designed as a process evaluation of the intervention with the use of both qualitative and quantitative methods. A local childhood health profile will provide data for childhood health issues, for example, daily physical activity behavior, sleep patterns, anthropometry, mental health, screen use, parental support, and leisure-time activities. Data at system level are collected to assess development in the community, for example, readiness to change, analysis of social networks with stakeholders, rippled effects mapping, and changes in system map. The setting is a small rural town in Denmark, Havndal, with children as the primary target group. Group model building, a participatory system dynamics method, will be used to engage the community, create consensus on the drivers of childhood health, identify local opportunities, and develop context-specific actions.

**Results:**

The Child-COOP feasibility study will test the participatory system dynamics approach for intervention and evaluation design and survey objective measures of childhood health behavior and well-being among the ~100 children (6-13 years) attending the local primary school. Community-level data will also be collected. We will assess the contextual factors, implementation of interventions, and mechanisms of impact as part of a process evaluation. Data will be collected at baseline, at 2 years, and 4 years of follow-up. Ethical approval for this study was sought and granted from the Danish Scientific Ethical Committee (1-10-72-283-21).

**Conclusions:**

s: The potential of this participatory system dynamics approach includes opportunities for community engagement and local capacity building to improve children’s health and health behavior, and this feasibility study holds the potential to prepare an upscaling of the intervention for effectiveness testing.

**International Registered Report Identifier (IRRID):**

DERR1-10.2196/43949

## Introduction

Children’s health and well-being are of great concern worldwide [1], with decreasing levels of physical activity, more sedentary time, emerging rates of childhood obesity, and more children living with mental health problems [1-5]. Childhood health is closely related to the socioeconomic status of the family, for example, children raised in families with low socioeconomic status more often have mental health problems, obesity, and a sedentary behavior [2,3,6]. Several studies suggest that it is possible to improve childhood health despite inequalities in health [2,3,7-10], and early interventions have shown to be important in childhood health promotion [11]. Childhood health is a result of a complex interplay between many factors at individual, family, and society levels. Hence, declining childhood health must be viewed as a complex problem and calls for multilevel interventions and broader societal awareness [12]. Within childhood obesity, interventions building on community-based participatory research, and system dynamics have shown promising results in addressing complex health problems of children [13] by involving whole of communities in complex problem-solving at multiple levels of actions. Researchers have highlighted such participatory and system dynamic approaches as a potential way forward [14,15], addressing complex health problems in communities [13,16].

However, before applying a full-scale participatory system dynamics intervention in Denmark, feasibility testing is needed to underpin decisions about whether or not and how to progress [16]. The 2021 Medical Research Council (MRC) framework for complex interventions stresses the importance of considering the phases of intervention development in an iterative manner, from development, testing, and evaluating to implementation of interventions. Moreover, it identifies important core elements such as context, engage stakeholders, and development of a program theory [16].

Building on Australian experience with the participatory system dynamics approach [13] and the MRC framework as guidance [14], this paper describes the Children’s Cooperation Denmark (Child-COOP) feasibility study design that is aiming to examine the feasibility and acceptability of the participatory system dynamics approach [17] and the study procedures intended for a future larger scale-up controlled trial.

## Methods

### Design

The Child-COOP feasibility study will apply a participatory system dynamics approach, and the MRC framework [16] informs the design of the feasibility study and guidelines for applying feasibility studies [18]. As suggested by the MRC, feasibility studies should be designed to assess the intervention and evaluation design.

### The Community

The Child-COOP feasibility study will be tested and evaluated in a small community in a rural town area (Havndal) with approximately 900 citizens, located in the northeastern part of a large Danish Municipality, Randers. Including the surrounding area, the local community consists of approximately 2000 citizens. The community holds an integrated school and kindergarten “The Child Village” with approximately 100 children in grades 0 to 6 and approximately 40 children in the kindergarten. A few shops such as a local fitness provider, leisure-time sport provider, and a relatively small business environment exist within the area.

### Populations

The target population is primary school–aged children (grades 0 to 6, 6-13 years of age; n=110), and all children attending the local primary school will be invited to participate in the health monitoring (described later in “Childhood Health: Outcome and Data Collection” section). Another target population is the key stakeholders of the local community, the local stakeholders, and municipality leaders and staff working within the community. These could include local politicians, grocery owner, chairperson of sports clubs, school board leader and members, local parents (with children at the school), leader of the municipality health department, and other municipality leaders, among others. The town of Havndal is one of the areas in Randers Municipality with the lowest social class and highest unemployment rate.

### Intervention

**Figure 1:**
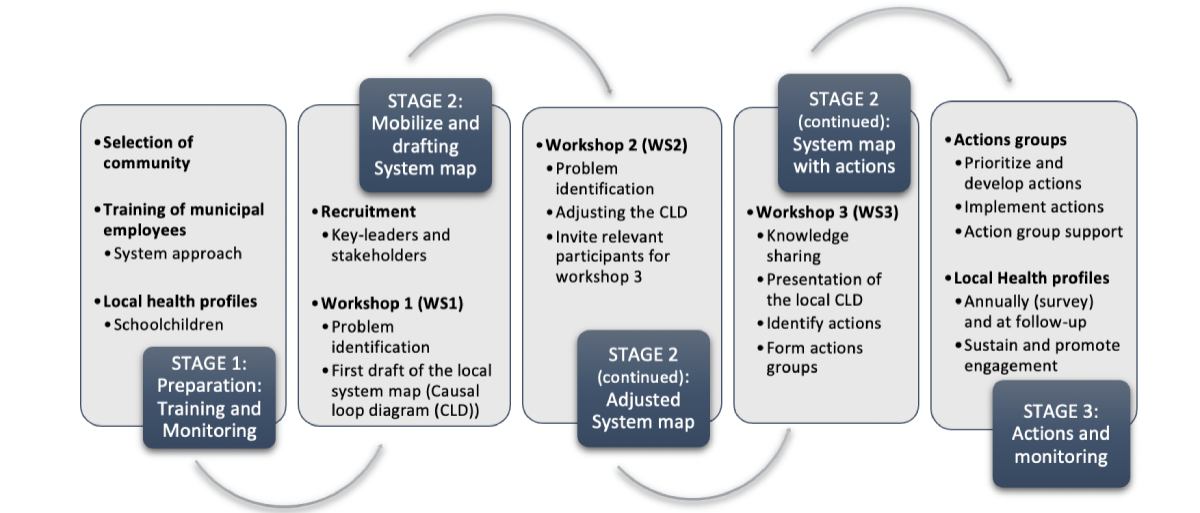
Illustration of the participatory system dynamics approach including the group model building processes. CLD: Causal loop diagram.

The Child-COOP feasibility study will apply a participatory system dynamics approach and consists of several elements illustrated in Figure 1 and described in the following sections.

#### Monitoring

A local childhood health profile will be generated at baseline, for example, daily physical activity behavior, sleep patterns, anthropometry, mental health, screen use, parental support, and leisure-time activities (Table 1).

**Table 1:** Outcome measurements at child level in the Children’s Cooperation Denmark feasibility study.

Concept	Outcomes of interest	Instrument or measure	Method	Data collection
Anthropometry	Height BMI Fat mass Prevalence of overweight	Tanita Leicester transportable height measure InBody 230 multifrequency body composition (bioelectrical impedance analysis)	Individual measurement	Examination at baseline and after 2 and 4 years
Physical activity and sedentary behavior	Minutes per day spent on moderate and vigorous physical activity and sedentary activity Minutes per day spent on Step counts	Accelerometer (Axivity AX3)	Individual measurement	7 days at baseline and after 2 and 4 years
Physical literacy	Motivation and confidence Physical competence Daily behavior Knowledge and understanding	A Danish version of the Canadian Assessment of Physical literacy	Individual measurement of physical competence (including motor skill test, aerobic capacity run, and torso strength test) and daily behavior (self-report weekly participation in moderate- to vigorous-intensity physical activity and objectively measured average step count for 1 week). Survey of motivation or confidence and knowledge or understanding.	Baseline and after 2 and 4 years
Mental health	General well-being Social relations—peers Loneliness Bullying Stress	Børnungeliv.dk	Survey	Baseline and after 2 and 4 years
Sleep	Hours of sleep Time of sleep week days or weekends Quality of sleep Use of electronic devices	Børnugeliv.dk and accelerometer (Axivity AX3)	Survey and individual measurement	Baseline and after 2 and 4 years
Leisure time	Participation in sport and other activities Use of computer, tablet, and other electronic devices	Børnungeliv.dk	Survey	Baseline and after 2 and 4 years
Body and movement	Active transport to school Time spent on physical activity in school Frequencies of high-intensity activity Physical fitness level Facilities for sports and play in the neighborhood Body satisfaction	Børnungeliv.dk	Survey	Baseline and after 2 and 4 years
Food and meals	Breakfast, lunch, and dinner Cooking at home Intake of fruit and vegetable, sugar-sweetened beverage, candy, and cake	Børnungeliv.dk	Survey	Baseline and after 2 and 4 years
Family relations	Siblings Country of origin (child and parents) Relation to parents Place of residence	Børnungeliv.dk	Survey	Baseline and after 2 and 4 years

#### Recruitment of Key Leaders and Local Stakeholders

A coordinator from Randers Municipality together with the research team identify and recruit key leaders (eg, local politicians, department heads, and municipality leaders) and local stakeholders (eg, school board members, school principal, school nurse, and sport club representatives). Key leaders and local stakeholders will be selected based on the authority and capacity to initiate actions that are likely to influence the children’s health behavior across sectors and organizations. During this stage, key leaders must commit to allocating resources to ensure subsequent implementation. The aim is to recruit 12-15 key leaders and local stakeholders from the community.

#### Group Model Building Process and System Mapping

A total of 3 group model building (GMB) workshops (WS1, WS2, and WS3) will be held during the 3 stages of the process (see Figure 1). The GMB method is an evidence-based method for solving complex challenges [13]. During WS1, the local childhood health profile will be presented, and based on this information, key stakeholders will discuss what health-related topic they find most important to their community (eg, obesity, physical activity, and mental health). In WS1 and WS2, the key leaders and stakeholders will map the system by creating a causal loop diagram (CLD) to understand how the perceived local system affects the prioritized childhood health topic in their community. For developing the CLD, the web-based software tool Systems Thinking in Community Knowledge Exchange (STICKE; version 3.0.14; Deakin University) was used [13]. STICKE stands for Systems Thinking in Community Knowledge Exchange and is developed to facilitate community knowledge exchange to foster shared understanding of complex problems.

In WS3, all community members willing to engage in changing the local system will be invited to identify priority areas for action based on the developed CLD from WS1 and WS2.

#### Actions and Support

The output of WS3 is the formation of local working groups that will focus on implementing the chosen actions using cocreation ideals [13]. The working groups will be supported and supervised by a backbone office formed by the Child-COOP municipality coordinator and the research team. A follow-up workshop will be held with the key stakeholders 6 months after the completion of WS3 to review the consolidated priority actions. To increase and maintain motivation and actions in the local community, subsequent follow-up meetings will be held with the working groups when needed after WS3.

### Assessment of the Evaluation Design

The feasibility study of the evaluation design for Child-COOP will involve multiple levels and perspectives with a focus on key aspects such as recruitment, data collection tools and processes, analysis, and unintended outcomes [16]. In Child-COOP, the evaluation design will include both quantitative and qualitative methods. The evaluation design includes (1) evaluation of childhood health and (2) evaluation of community readiness to change, social network, actions implemented, and system changes.

Child-COOP will be considered feasible if it meets the following criteria: (1) The GMB sessions were conducted as planned. (2) Working groups are formed and active in implementing interventions arising from GMB. (3) Outcome measures were collected and considered complete (>75% considered acceptable at baseline). (4) Response rate considered acceptable (>75% considered acceptable at baseline).

### Childhood Health: Outcome and Data Collection

#### Overview

All measurement instruments are listed in Table 1 and briefly explained. The measures will be conducted over 2 test days at the local school to assess the health of the children and test the data collection procedure. On day 1, children in grades 4, 5, and 6 are invited to participate, and on day 2, those in grades 0, 1, 2, and 3 are invited to participate. All children available on the day of data collection, whose parents have given written consent, will be included. A team of trained data collectors will collect the data on the test days with assistance from the class teachers. The longitudinal design makes it possible to study the changes over time within the local community, with limited effectiveness testing due to the limited power, as this is a feasibility study.

#### Anthropometry

All included children will have height, weight, and fat mass measured, wearing light clothes and barefoot, by the school nurse (Table 1).

#### Physical Activity and Sedentary Behavior

Physical activity and sedentary behavior will be measured continuously over a 7-day and -night period using Axivity AX3 accelerometers (Table 1). The accelerometer will be attached to the skin on the medial front of the right thigh using skin tape. The procedure is described in detail elsewhere [19]. The accelerometer is water-resistant, and the skin tape is intended to be water-resistant; thus, the accelerometer can be worn when swimming and bathing. The OMGui software (version 1.0.0.43; GitHub Inc), which is available on the internet, will be used for instrument initialization and data download [20].

#### Physical Literacy

The Danish version (DAPL) of the Canadian Assessment of Physical Literacy [21] will be used to measure physical literacy for children in grades 1 to 6 [22]. Grade 0 children will not be included in the assessment tool and have neither been used nor validated, as we consider these children too young for the assessment [22].

#### Self-reported Health and Well-being

The children will fill a questionnaire at school with assistance from their teacher and trained data collectors, more questions for children in grades 4 to 6 and less for children in grades 0 to 3. The parents will also fill a questionnaire about their child’s health and well-being with more questions for children in grades 0-3 and less for children in grades 4-6.

We will use a Danish-validated questionnaire instrument (Danish: BørnUngeLiv, English: ChildYouthLife) developed by researchers and practitioners from the municipalities to use in the municipalities of Denmark to assess the health and well-being of children [23]. Topics that will be covered in the questionnaires are mental health, sleep, leisure-time activities, body and movement, food and meals, and family relations (see Table 1).

### System Level: Outcomes and Data Collection

Data at system level will be collected continuously from baseline and with 2- and 4-year follow-up. All outcomes and measurement instruments are listed in Table 2 and briefly explained.

**Table 2:** Community outcome measurements in the Children’s Cooperation feasibility study.

Item	Outcomes of interest	Instrument or measure	Method	Data collection
Readiness to change	Baseline and change in: Community knowledge about child obesity Existing community efforts Community knowledge of the efforts Leadership Community attitudes Resources related to child obesity	The community readiness model	One-to-one interviews	12 interviews of 45-75 minutes Collection before WS1^a^ 1-year follow-up
Social network	Strength and importance of relationships Density Centrality “Opinion leader” And change over time	Social network dynamics	Survey Semistructured interviews	Survey delivered to stakeholders Collection before WS1 1-year follow-up 2-year follow-up
Actions implemented	Number of actions implemented Place of influence within the CLD^b^ Stakeholders	REM^c^ Add action variables within system dynamics software (STICKE^d^)	Ongoing communication with working groups after WS3^e^ Semistructured interviews with working group leaders	Using REM as a method to track actions developed 1-year follow-up 2-year follow-up 4-year follow-up
Change in system maps	CLD	CLD using STICKE	Monitoring CLD Focus groups Adjustment of CLD	CLD at WS3 Collection of focus groups at 2-year follow-up 4-year follow-up Based on focus groups

^a^WS1: workshop 1.

^b^CLD: causal loop diagram.

^c^REM: rippled effects mapping.

^d^STICKE: Systems Thinking in Community Knowledge Exchange.

^e^WS3: workshop 3.

#### Readiness to Change

To assess whether changes occur in the community’s readiness and capacity for making system changes, we use the community readiness model [24] forward-and-back translated to Danish [25]. Community capacity and readiness here refer to a “community’s ability to identify, mobilize and address public health problems” [26]. Responses will be used to measure the changes in community capacity by scoring the descriptive responses on anchored scales [24,25]. Data will be collected from key leaders in the community including school principal and staff, local government leaders, parent representatives, among others. It is hypothesized that Child-COOP will change the readiness to change in the local community.

#### Social Network Analysis

Social network analysis will be used to assess the development of relationships between people and organizations [27]. Social network analysis can calculate network statistics including density and centrality and “opinion leader” positions [13]. Furthermore, social network dynamics will be applied with longitudinal network models in mind to determine how the networks change over time and the role of the network in the diffusion information, knowledge, and practice [28]. Data will be collected using the COMPACT Stakeholder-driven Community Diffusion Survey [29] adapted to a Danish context. It is hypothesized that Child-COOP will change and connect more people within the community’s social networks.

#### Actions Implemented

Proxy indicators of system change will be actions initiated by the community, and these will be monitored from baseline and followed up annually using the STICKE software [30]. The number of community actions will be tracked as proxies of community-level engagement [13]. Semistructured interviews with working group leaders will also be used to understand and provide deeper information on the actions implemented (eg, goal, content, and setting) [31]. To track the actions developed and implemented in the local community, we will use “ripple effect mapping” (REM) [32]. REM is a method used to better understand the complex, dynamic nature, and wider impacts of system dynamics intervention [33]. Unlike traditional evaluation designs, REM is aimed at understanding contribution; how may an intervention, action, or policy contribute toward changing a larger system? [32-34] During the workshops, participants (stakeholders, citizens, municipality employees, etc) visualize the impacts of the actions and how these impacts may go beyond those, which Child-COOP was designed to achieve [32].

#### Change in System Map

Tracking of change in the CLD and the communities’ responses to the participatory system dynamics approach will be monitored through a revised system map at 2 and 4 years post the initial CLD [13]. The STICKE software [30] will be used to track changes in the system and the interaction among key actors, actions, and the system.

### Assessment of the Intervention: Process Evaluation

The process evaluation aims to understand the functioning of an intervention by examining mechanisms of impact, implementation, acceptability, and context.

Through analyses, crosscutting qualitative and quantitative results on the implementation (adherence, dose, quality of delivery, participant responsiveness, and reach), mechanism of impact, acceptability, and context will be synthesized to gain knowledge on how the participatory system dynamics approach was delivered and implemented and inform a final program theory.

#### Mechanism of Impact

**Figure 2:**
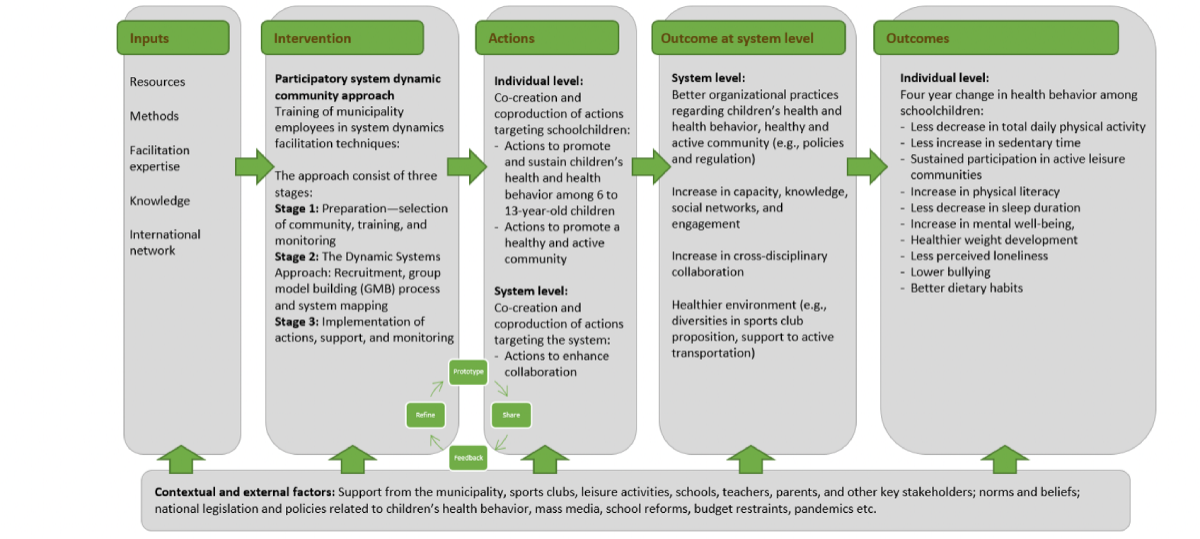
Child-COOP tentative program theory for feasibility study in Havndal, Denmark. Child-COOP: Children’s Cooperation Denmark.

What are the change mechanisms for Child-COOP and how do they align with program theory? These questions will be addressed through 12 semistructured interviews with key stakeholders and the municipal coordinator, addressing the research questions: *What are the mechanisms of change associated with the participatory system dynamics approach? What hinders or enables implementation of the participatory system dynamics approach?* Our program theory is a tentative developed program theory for the overall study (see Figure 2), while the developed CLD will be considered a form of logic model [16].

#### Implementation

What is delivered in Child-COOP? Here, we aim to understand how the “implementation” is done locally in the community assessing (1) fidelity (is the participatory system dynamics approach delivered as intended?); (2) dose (who is reached by the participatory system dynamics approach and to what strength?); (3) adaptions (have any significant changes to the participatory system dynamics approach been made?); (4) reach (how many are part or affected by the participatory system dynamics approach?); and (5) process (understanding the implementation process?). These elements are sought to understand through structured observations by registration and participation (number of participating stakeholders or established working groups or initiated actions) and a questionnaire for stakeholders.

#### Acceptability and Context

How is “acceptability” of the Child-COOP approach among the participants? Through 12 semistructured interviews with key stakeholders and participants, we will collect data aiming to understand the following: *How do participants involved in workshops and implementation of actions react to and engage in the Child-COOP approach?* Furthermore, we aim to conduct follow-up phone interviews with dropout key stakeholders to provide a realistic perspective.

### Ethics Approval

This study will be carried out in accordance with the Declaration of Helsinki. Ethical approval for the study has been granted by The Regional Scientific Ethics Committee of the Central Denmark Region, Danish National Committee on Health Research Ethics (1-10-72-283-21). Any protocol amendments will be reported and submitted to the Ethics Committee. Anonymity and confidentiality of participants will be ensured by assigning a study ID number to all participants. Informed consent will be obtained from parents in order to include the children in the health assessments. No results of the anthropometric measurements will be visible to, or available for, the children to reduce risk of the bullying and stigmatizing.

### Consent for Publication

Informed consent will be collected from parents on behalf of the children taking part in the study. Informed consent will be collected from all participants in the workshops.

## Results

The Child-COOP feasibility study has officially started with baseline measures in September 2021, recruitment of participants and delivery of the GMB phase started in 2021-2022. The 2-year follow-up will be the next step and will reveal the first results of the study—this is expected in 2023. Furthermore, process evaluation is underway and will be concluded at the 2-year follow-up in 2023. The project will conclude with the 4-year follow-up, which will provide the final results—these are expected in 2025. In September 2022, a large grant was given for the Child-COOP Denmark project, focusing on children’s physical activity behavior. This project will include 5 municipalities and 10 communities, building on the experiences of the Child-COOP feasibility study. It will begin immediately after the conclusion of the process evaluation of the Child-COOP feasibility study in late 2023 or early 2024.

## Discussion

### Expected Findings

The Child-COOP feasibility study will provide new knowledge on the potential to implement a participatory system dynamics approach targeting childhood health in a Danish context. Childhood health is a complex health problem and evidence points to system science as one of the best means of identifying and addressing such complex and dynamic problems [13]. The approach will be tested out in a small disadvantaged community in Denmark, and therefore, it may be applicable in similar settings upon a positive feasibility assessment.

### Strengths of the Study Design

Interventions building on a participatory system dynamics approach as Child-COOP have already been suggested as a feasible way to address complex problems, as they combine the current evidence base on prevention, best practice, and local wisdom to achieve new knowledge and create solutions [16,34]. The participatory system dynamics approach in Child-COOP based on the co-creation and close collaboration between the municipality, community, and facilitating research team will most certain be a strength of the Child-COOP feasibility study. In addition, this study builds upon existing evidence and shows a positive effect of a participatory system dynamics approach in improving the childhood health and obesity rates [13]. Furthermore, to investigate the complex relationship of childhood health drivers in the community, the active involvement of the local community in mapping, developing action plans, and maintaining the efforts may provide sustainability and empower the local community [13]. The extensive evaluation design using multiple methods for data collection at the individual level as well as the system level also serve as a strength of this study.

### Potential Challenges

Potential challenges of the study include the long-term engagement of the local community, which may decline over time; however, this feasibility study will provide insight into the magnitude of the support from the research team, local leaders, or coordinators to develop, facilitate, and support the working groups to continue to implement and adjust actions in their local community. Moreover, a potential challenge is that the community will not take the chance to change and influence the local system affecting the health and well-being of the children.

In addition, external factors may challenge the implementation of the intervention, for instance, the COVID-19 pandemic or local and municipality economic challenges. However, the participatory system dynamics approach is flexible and can be adapted to be conducted largely on the internet while still representing the local context. Finally, we acknowledge that a whole of system approach for evaluation may never be fully achievable, as changes in a complex system will always give rise to more uncertainties than a single evaluation can satisfactorily capture [34,35]. However, with our feasibility assessment, we focus on the most important areas of uncertainty and to justify decisions of which assessment of impact can be expected to be meaningful at a system level.

### Conclusions

In conclusion, the Child-COOP feasibility study is crucial in evaluating the potential for a full scale-up study in a Danish context. If feasible, this participatory system dynamics approach in Child-COOP provides opportunities for the application of local capacity building by applying a practical approach to complex health problems in a local community [34]. This study will be able to inform both the content of the participatory system dynamics approach and the future larger-scale evaluation design. Furthermore, the community outcomes included may help to better understand the changes in the community and the mechanisms leading to changes in childhood health within communities [13]. If proven feasible, the Child-COOP study will be scaled and tested for the effectiveness in relation to improving childhood health in a Danish context.

## Abbreviations

Child-COOP: Children’s Cooperation Denmark
CLD: causal loop diagram
GMB: group model building
MRC: Medical Research Council
REM: ripple effect mapping
STICKE: Systems Thinking in Community Knowledge Exchange
